# Study of Thixotropic Characteristics of a Kerosene Gel Propellant by Bayesian Optimization

**DOI:** 10.3390/gels9010015

**Published:** 2022-12-26

**Authors:** Hao Zhou, Cai Chen, Feng Feng, Changsheng Zhou, Wenling Zhang, Wei-Tao Wu

**Affiliations:** 1 School of Mechanical Engineering, Nanjing University of Science & Technology, Nanjing 210094, China; 2 Sino-French Engineer School, Nanjing University of Science and Technology, Xiaolingwei 200, Nanjing 210094, China

**Keywords:** kerosene gel propellant, thixotropic, Bayesian optimization

## Abstract

The rheological behavior of gel propellants is crucial for their practical applications, especially in the rocket engine and ramjet fields. The thixotropic characteristics of gel propellants are an important component of their rheological properties and have a notable impact on their flow and injection process. However, most gel propellants contain rich, dynamic cross-linked network structures, which impart complex non-Newtonian fluid properties, and it is difficult to establish a unified mathematical model. In view of this, this study addresses the thixotropy of a prepared RP-3 kerosene gel and determines the mathematical model and model parameters describing its thixotropy. Experiments show that the kerosene gel exhibits shear-thinning properties as well as thixotropy. To describe the microstructural changes in the gel, three thixotropic constitutive models are introduced to analyze the rheological data, and the constitutive equation parameters are optimized. The three models are all structural dynamic models, which can be used to describe microstructural changes within the material. In addition, the fitting of the constitutive equation is a multiparameter optimization problem, and an appropriate optimization method must be used for parameter fitting. Therefore, the Bayesian optimization method combined with Gaussian process regression and the upper confidence bound (UCB) acquisition function is used in the multiparameter fitting of the constitutive models. Both experiments and numerical results show that the thixotropic model, which introduces a pre-factor with shear strain and assumes that the breakdown of the gel structure is related to energy dissipation rather than the shear rate, has a better fitting effect and prediction ability with regard to the gel. Combined with transient experiments at different shear rates, the model parameters of the constitutive law can be determined quickly by applying the Bayesian optimization method.

## 1. Introduction

With the increasing demand for rocket and ramjet propellants that provide good performance and safety, gel propellants have become a focus of research in recent decades. These propellants combine the advantages of liquid and solid propellants [1,2,3]. For instance, gel propellants can avoid the cracks and leaks that occur in solid or liquid propellants under harsh conditions, such as vibration, overload and impact [4,5]. Moreover, gel propellants have a higher specific impulse and wider thrust adjustment capability than solid propellants [6,7], which makes them more attractive in both tactical rockets and space application rockets.

A gel propellant is a gel system formed by adding a gelling agent to a liquid propellant. The gelling agents are combined into a three-dimensional network gel structure through noncovalent intermolecular interactions [5,8,9,10,11]. In this paper, the noncovalent interaction of the gelling agent (thixatrol ST) is H-bonding. Due to the existence of the network structure, the original liquid propellant is mixed into the gel network, which inhibits the flow and forms a gel structure, and the gel exhibits elasticity at this time. A continued increase in the shear stress disrupts the gel structure, decreasing the viscosity and imparting strong fluidity [4,8,12]. The gel exhibits strong non-Newtonian fluid properties due to its complex microphysical structure, which makes the associated rheological properties difficult to describe. The rheological properties of the gel propellant have a strong influence on its flow and spray processes. Therefore, the description and evaluation of the rheological properties of the gel is important for practical applications.

The gel propellant has thixotropy due to its intermolecular network structure. Thixotropy is one of the most challenging problems in the field of colloidal rheology. Schalek et al. [13,14] pioneered the phenomenon of thixotropy in a gel composed of an ${\mathrm{Fe}}_{2}O_{3}$ aqueous dispersion, which could be transformed reversibly into a liquid solution through shaking under isothermal conditions. Coussot et al. [15] developed a colloidal system using a bentonite suspension to investigate viscosity bifurcation in thixotropic and yielding fluids. Pramanik and Zanna et al. [16,17] investigated the application of thixotropic hydrogels in the biomedical field. All the above studies show that the viscosity of thixotropic fluid is related to its shear history. At a constant shear rate, the viscosity of the gel decreases with time. This differs from the common shear thinning property of gels, where the viscosity of the gel decreases as the shear rate increases. At a constant shear rate, the viscosity does not change. However, both thixotropy and shear thinning properties of the gel affect the injectable properties of the gel. The lower the viscosity of the gel, the more injectable it is. In the case of the engine, the injectability is reflected in the nozzle. At the same pressure drop, the lower the viscosity, the better the injection at the nozzle obtained and the better the atomization effect achieved. Therefore, thixotropy and dilution characteristics affect the flow and atomization characteristics of the gel, and then affect the working process of the engine.

The rheological properties of different gel propellants have been popular in recent decades. NASA’s Lewis Research Centre has carried out rheological property and stability research based on the RP-1 gel propellant and metallized gel propellant Al/RP-1 [4]. Their research shows that the above gel exhibits shear thinning and thixotropy and that the yield stress of the metallized gel increases with increasing metal particle concentration. Rahimi et al. [18,19] selected different fuels and additives to configure different gel fuels, mainly studied the rheological characterization of the prepared gel and the influence of temperature on the gel simulation liquid, and confirmed that the Herschel–Bulkley constitutive model has a good fitting effect on the prepared gel. Madlener et al. [20,21] used paraffin, kerosene and ethanol as base fluids and prepared gel fuels with different gelling agents and additives and used the extended Herschel–Bulkley model to describe the associated rheological properties. Gupta et al. [22] used the Casson model to fit the yield stress value of a fuming nitric acid gel. Dennis et al. [23] measured the rheological properties of monomethyl hydrazine gels and fitted the gel rheological parameters by using a power-law model. Arnold et al. [24] formulated JP-8 and RP-1 fuel gels by using fumed silica as a gelling agent and studied their apparent viscosity, stability and thixotropy. Jyoti et al. [8] measured the rheological properties of metallized and nonmetallized ethanol gels with different gelling agent contents and provided the rheological parameters under the power-law model. Santos et al. [25] qualitatively analysed the thixotropy of silica hydrocarbon gels.

Generally, thixotropic models can be classified into three categories: phenomenological models, microstructure models and structural kinetic models [10,12]. By comparison, the structural dynamics model can effectively reflect the microstructural changes in gels to a certain extent without in-depth understanding of microstructural changes and is most widely used in thixotropic modelling research [26]. Cheng et al. [27] proposed a general form for modelling thixotropy by using a structural kinetic approach. On this basis, Dullaert et al. [28,29] used fumed silica and carbon as dispersed phases to successfully configure a thixotropic fluid system. After measuring its rheological properties, a structural kinetic model was proposed for the thixotropic system. Rahimi et al. [30] used a kinetic model proposed by Tiu et al. [31] to describe the thixotropy of inorganic silica gels. Negrão et al. [26] proposed a structural kinetic model to simulate the start-up flow of drilling fluid in a drill pipe. Li et al. [32] used a time-term-based model to describe the thixotropy of gum arabic. Rooij et al. [33] developed a microrheological model for the steady shear behaviour of weakly aggregating dispersions that combined the concept of fractal aggregation in shear flows with transient network modelling originally developed for polymer dynamics. The validity of the model was tested by fitting it to experimental data on a well-characterized weakly aggregating polystyrene latex dispersion.

Kerosene gel propellant with the addition of metal particles could be used for increasing the specific impulse of the rockets, however, this will be addressed in the next study. In this work, the kerosene gel propellant without the addition of metal particles is considered because of the following reasons: (i) With the addition of metal particles, the characteristics of kerosene gel is more complicated; moreover, metal particles are flammable or toxic; as a result, the research process safety requirements are reduced, which is not easy to realize; (ii) the mechanical properties of kerosene gel are complex, and the research process of thixotropy requires a large number of experiments to obtain stable and accurate experimental data, and then most of the theoretical models need to fit to get a reliable model parameter. By a study on relatively safe kerosene gel without metal particles to carry out system mechanics research, we constructed the research methods for kerosene gel mechanical property measurement, modelling and model fitting, which provide a solid foundation for the research of kerosene gel containing metal particles, thus shortening the corresponding research time in future; (iii) organic kerosene gel without metal particles also has application in some rocket propulsion systems, such as a kerosene gel ramjet engine, in which kerosene gel is used to replace kerosene to improve the safety of the equipment during transportation, storage and operation.

The rheological properties of gel propellants have been widely studied both numerically and experimentally; however, there have been few studies on their thixotropic properties, and only qualitative analysis of the thixotropy of gel propellants has been carried out. Therefore, this study focused on the thixotropy of gel propellant, and quantitatively analysed its thixotropy characteristics through experimental measurement and three different structural dynamic thixotropy models. Combined with the Bayesian optimization method, the mathematical model and model parameters describing the thixotropy could be quickly determined. Subsequently, the researchers configured various gel systems containing thixotropy for theoretical and applied research on thixotropy. Coussot et al. developed a colloidal system using a bentonite suspension to investigate viscosity bifurcation in thixotropic, yielding fluids. Pramanik and Zanna et al. investigated the application of thixotropic hydrogels in the biomedical field.

## 2. Results and Discussion

### 2.1. Experiment and Discussion

The fitting results are presented and discussed in this section. First, some parameters in the equations are determined according to the experimental measurement results. Then, combined with the transient measurement results, all the remaining parameters in the constitutive models are provided by using the numerical fitting method. The obtained parameters are brought into the equilibrium equation to obtain an equilibrium viscosity curve and test its fitting effect on the equilibrium viscosity value. Both the transient viscosity fit results and the equilibrium viscosity fit results are used to evaluate the model fit ability. Finally, the prediction ability of the models is verified by the viscosity change curve of the gel under the step–shear rate.

**Figure 1:**
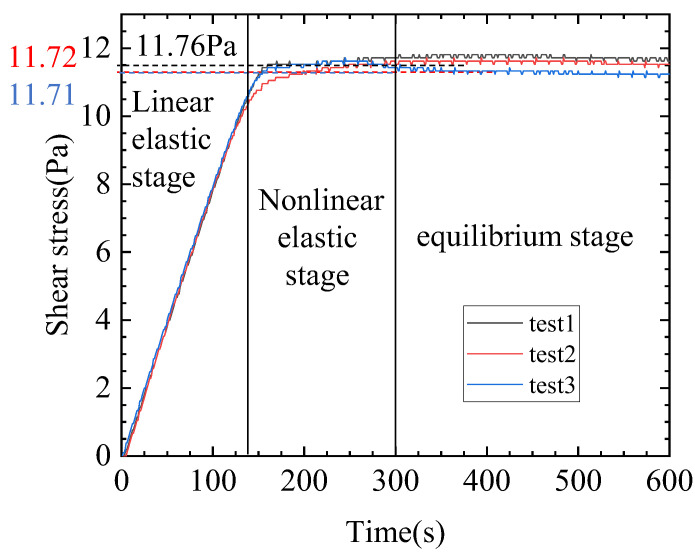
Comparison of the three yield stress measurements.

The yield stress of the gel material is obtained by directly measuring the stress–time curve, as shown in Figure 1. The three curves are in good agreement, and the average of the maximum values of the three curves is $\tau_{y}$= 11.73 Pa.

For the Toorman model, its parameter C can be obtained from the hysteresis loop curve. When the gel is in the pre-yielding state, it can be considered that its structural parameters remain constant with λ= 1. As the gel starts to flow, λ starts to decrease from 1.

When λ=1, the following relationship can be derived from the Toorman model:

(1) $$\frac{d\tau }{d\dot{\gamma }}=\eta_{\infty }+C$$

which describes the average slope of the hysteresis loop curve in the initial stage. The value of C is 22.06. The experimental results and parameter fitting results are shown in Appendix D.

**Figure 2:**
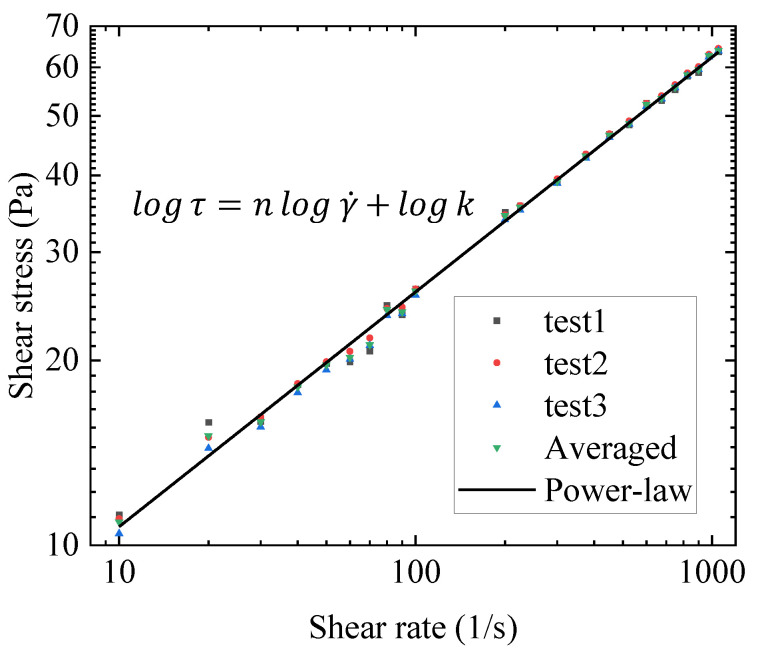
Flow curves of the shear stress vs. shear rate after high-speed shearing.

After the gel is sheared at high speed, the stress–shear rate relationship is measured. The results are shown in Figure 2.

After high-speed shearing, the gel behaves as a power-law fluid, and the power-law model can be used to fit the stress–shear rate relationship. The fitting process is shown in Appendix E, and the fitting results are good.

For the Houska model and the Teng model, as the structural parameter λ approaches 0, the thixotropy and yield stress disappear, and the state equation can be simplified into a power-law model. This is consistent with the experimental phenomenon after high-speed shearing. The hysteresis loop experiment of the gel after high-speed shearing is described in Appendix E. Therefore, k and $n_{1}$ in the Houska model and Teng model can be determined by the fitting results of this experiment as k = 4.14 and $n_{1}\;$= 0.395, respectively.

### 2.2. Numerical Fitting Results and Discussion

For the Toorman model, Houska model and Teng model, the remaining parameters are obtained by fitting the transient viscosity change using the Bayesian optimization method. The optimization objective is the mean relative error (MRE). In the transient process of fitting, the stage before yield is ignored, and the fitting starts from the maximum point of the viscosity–time curve at t = 5 s. Since the Bayesian optimization process is a high-dimensional “black box process” in this problem, it cannot be visually analysed. Therefore, only the fitting results and part of the two-dimensional visual fitting process are provided. The remaining parameters and the average relative error are shown in Table 1.

**Figure 3:**
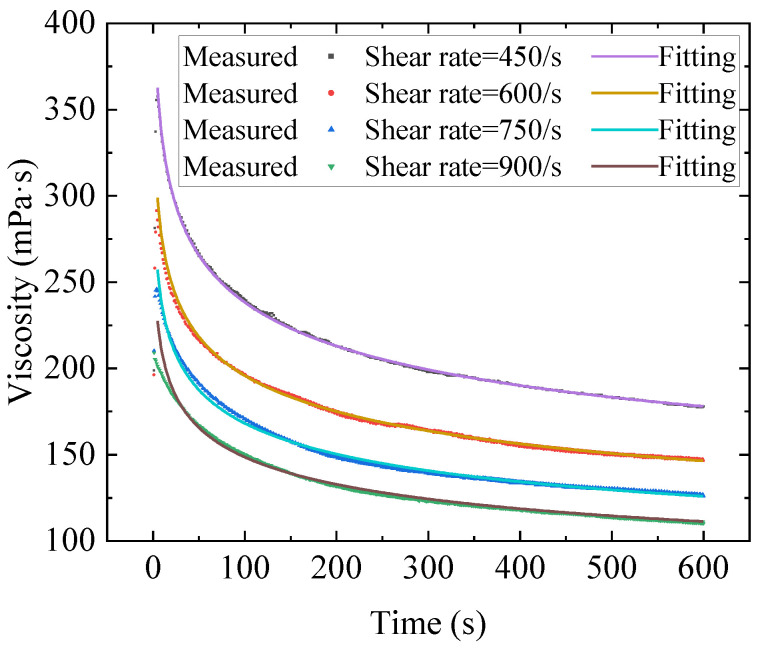
Fitting lines of the transient viscosity using Teng model.

The Teng model has the best fitting effect, and the fitting results are shown in Figure 3. The transient process fitting results of other models are shown in Appendix F, as is the fitting of parameters to the equilibrium equation. The Teng model can not only fit the transient change process of the gel well but also provides a better fitting result for the steady-state viscosity.

**Figure 4:**
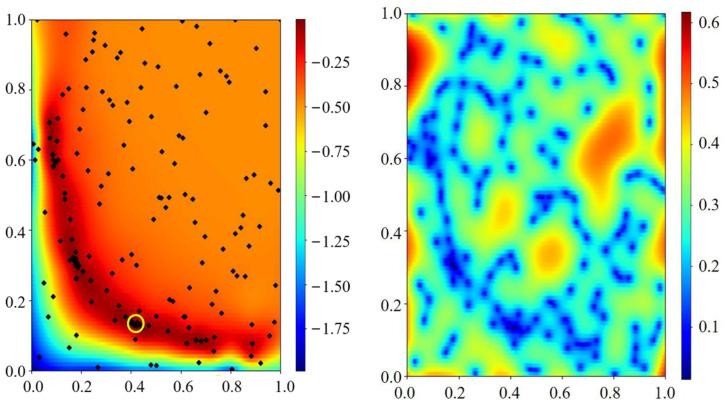
Two parameter optimization process of a and b in the Teng model. The horizontal axis is the a/b with normalization, and the longitudinal axis is the b with normalization.

Figure 4 shows the two-dimensional visualization fitting results of parameters a and b in the Teng model; the parameter optimization ranges are as follows: b∈(0,0.1], a/b∈ [0,2]. The left figure shows the optimized mean, and the right figure shows the variance, where the parameter range has been normalized. The optimization process finally converges to a/b= 0.917 and b= 0.0109. The black dots in the figure are accurately calculated sampling points. In the case of small sampling, the solution process can jump out of the local optimal solution minimum, such as the blue circle in Figure 4, and finally converge to the global optimal solution shown in the yellow circle. This shows that the Bayesian optimization method provides good global convergence. The rest of the two-parameter visualization process can be found in Appendix G.

**Figure 5:**
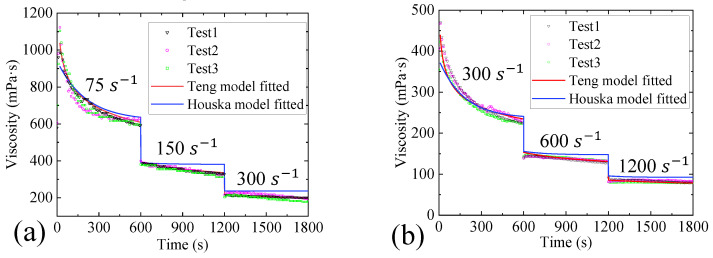
Measurement and prediction curve of the gel viscosity change under the step–shear rate under viscosity of (**a**). 75 s^−1^, 150 s^−1^ and 300 s^−1^; (**b**). 300 s^−1^, 600 s^−1^ and 1200 s^−1^.

To further evaluate the predictive ability of the models, the obtained model parameters were compared with the step–shear rate curves obtained in the experiments. Since the Toorman model has a poor effect on the transient fitting of the gel, only the predictive ability of the Houska model and the Teng model is considered. In Figure 5a, the shear rates used in the step–shear rate curve are 75 $s^{-1}$, 150 $s^{-1}$ and 300 $s^{-1}$. In Figure 5b, the shear rates used in the step–shear rate curve are 300 s^−1^, 600 $s^{-1}$ and 1200 $s^{-1}$. The fitting results are shown in Figure 5.

From the prediction results, both the Teng model and the Houska model have a certain predictive ability for the viscosity change in the gel under the step–shear rate. However, the Teng model performs better than the Houska model. Therefore, it can be considered that the Teng model combined with the parameters obtained by the Bayesian optimization method in this paper has good predictive ability for the gel.

## 3. Conclusions

In this study, kerosene gel was prepared by using kerosene, Thixatrol ST and absolute ethanol. Then, the rheological properties of the gel were measured by using a rotational rheometer. Finally, the rheological properties were fitted and analysed by combining three constitutive equations and the Bayesian optimization method. Based on the investigation, the following conclusions and prospects can be drawn:

(1) Using kerosene, Thixatrol ST and absolute ethanol, the kerosene gel system was successfully prepared through water bath heating and high-speed dispersion. The system features yield, shear thinning and thixotropic properties.
(2) The kerosene gel is a plastic fluid with a yield stress of approximately 11.73 Pa. The yield stress was generated by the formation of a network structure through hydrogen bonding between the gelling agents. Upon high-speed shearing, the yield stress disappeared due to the destruction of the network structure.
(3) The gel has shear thinning and thixotropic properties. Hysteresis loop experiments indicated that the apparent viscosity of gels without high-speed pre-shearing decreased with an increasing shear rate. In the rising and falling stages of the shear rate, the gel viscosity showed a prominent time dependence, that is, thixotropy. The high-speed pre-shearing gel exhibited only shear-thinning properties. The power-law model had a good fitting effect on the rheological properties of the gel after high-speed shearing.
(4) The Bayesian optimization method can effectively fit multiple parameters at the same time. The method has the ability of global search and local optimization, which can effectively prevent the fitting process from falling into a local optimum.
(5) Three constitutive models were used to fit the rheological properties of the gel by combining with the Bayesian optimization method. The results show that the fitting of the Toorman model failed completely, the Houska model had a certain fitting and predictive ability, and the Teng model performed the best. Moreover, the Teng model effectively modelled the thixotropy of the gel and has a strong predictive ability.

The work in this paper provides a good beginning for our future research on kerosene gel. In future work, we will further consider the effects of temperature and the addition of metal particles on the gel’s constitutive model and parameters. At the same time, all the constitutive models used in this paper are viscoplastic models, which do not consider the elastic effect of the gel. In many situations, the elasticity of gel cannot be ignored and affects the flow and atomization of gel fuel flow greatly; therefore, the elasticity will be one of the key research contents in future. More significantly, it is critical to revealing the viscosity impact on rheological behaviour of gellant fluid arising from the different Newtonian base fluids, such engine oil, etc., which will be exploited in the near future.

## 4. Materials and Methods

The kerosene gel was prepared in the laboratory via the fabrication method described in Appendix A. The related experiments were carried out on the prepared kerosene gel for rheological property investigations, which included a yield stress measurement, transient viscosity change measurement, gel equilibrium viscosity measurement, hysteresis loop experimental measurement and rheological property measurement of the gel after high-speed shearing. The experimental conditions and measurement process are shown in Appendix B.

### 4.1. Thixotropy Model

In this paper, three structural dynamic constitutive models, the Toorman model, Houska model and Teng model [34,35,36], were used to fit the rheological properties of the prepared gels.

The model proposed by Toorman is shown in Equations (2) and (3).

(2) $$\tau =\lambda \tau_{y}+\left(\eta_{\infty }+C\lambda \right)\dot{\gamma }$$

(3) $$\frac{d\lambda }{dt}=a\left(1-\lambda \right)-b\lambda \dot{\gamma }$$

Here, λ is the structural parameter of the material and varies from 0 to 1; a, b, C are all constant terms; and $\eta_{\infty }$ is the viscosity of the material at an infinite shear rate. In addition, a(1−λ) is the build-up term of the material structure, and $b\lambda \dot{\gamma }$ is the breakdown term of the material structure. As the shear rate $\dot{\gamma }$ approaches infinity, the gel’s viscosity approaches a constant. For kerosene gel, it can be considered that the microstructure of the material is completely disrupted at an infinite shear rate, and its infinite shear viscosity can be approximately considered as the viscosity of kerosene.

The Houska model was a model proposed by Houska for the rheological properties of slurries of solid objects. The model is described through Equations (4) and (5):

(4) $$\tau =\tau_{y0}+\lambda \tau_{y}+\left(k+\Delta k\lambda \right){\dot{\gamma }}^{n_{1}}$$

(5) $$\frac{d\lambda }{dt}=a\left(1-\lambda \right)-b\lambda {\dot{\gamma }}^{m}$$

where $\tau_{y0}$ is the constant component of the yield stress; $\lambda \tau_{y}$ is the yield stress value of the structure; $a,b,\;m,n_{1},\Delta k,k$ are all constants; a(1−λ) is the build-up term of the material structure; and $b\lambda {\dot{\gamma }}^{m}$ is the breakdown term of the material structure. Regarding the gel prepared in this paper, upon high-speed shearing, the gel exhibited only fluidity. Thus, the constant component of the yield stress $\tau_{y0}$ can be ignored as approximately $\tau_{y0}=0$.

Teng et al. [36] proposed a thixotropic model, as shown in Equations (6) and (7), when studying the thixotropy of waxy crude oil:

(6) $$\tau =\tau_{y0}+\lambda \tau_{y}+\left(k+\Delta k\lambda \right){\dot{\gamma }}^{n_{1}}$$

(7) $$\frac{d\lambda }{dt}=\frac{1}{1+\gamma^{n_{2}}}\left[a\left(1-\lambda \right)-b\lambda \phi^{m}\right]$$

The definitions of $a,\;b,\;m,\;n_{1},\;\Delta k,\;k$ are the same as those of the Houska model, and they are all constants. In addition, ϕ is the rate of the energy dissipation, and for simple flows, $\phi =\tau \dot{\gamma }$. When using this model, the constant value $\tau_{y0}$ of the yield stress can also be ignored, approximated by $\tau_{y0}=0$.

### 4.2. Numerical Fitting Method

In this paper, the parameters in the above models that cannot be directly or indirectly obtained through experiments are obtained by the multiparameter fitting method—Bayesian optimization method. The Bayesian optimization method can fit the sample space through a small number of initial sample points and reasonable exploration methods.

The Bayesian optimization method mainly consists of two parts. One is the surrogate model. The second is the acquisition function used to guide the optimization direction. When using Bayesian optimization to solve practical problems, it is important to select appropriate probabilistic surrogate models and acquisition functions. However, with regard to Bayesian optimization, there are no unified and clear selection criteria for surrogate models and acquisition functions. Moreover, this approach still relies on manual selection. In this paper, the surrogate model uses a Gaussian process model. The acquisition function is the upper confidence bound (UCB). The initial samples were generated by using the Latin hypercube sampling method. The pseudocode, optimization model and principles of the sampling method are shown in Appendix C.

## Figures and Tables

**Table 1:** Fitted model parameters of the proposed model.

Model	Δk	$n_{2}$	m	a	b	C	Steady-State MRE	Transient MRE
Toorman	-	-	-	0.729	0.162	22.06	87.2%	10.9%
Houska	6.57	-	0.20	0.0024	0.0010	-	19.1%	1.8%
Teng	17.54	0.73	0.60	0.010	0.0109	-	6.9%	0.58%

**Figure A1:**
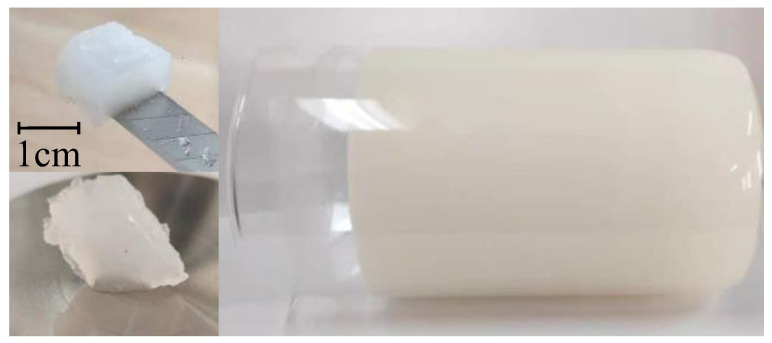
Example of the gelled fuel.

**Figure A2:**
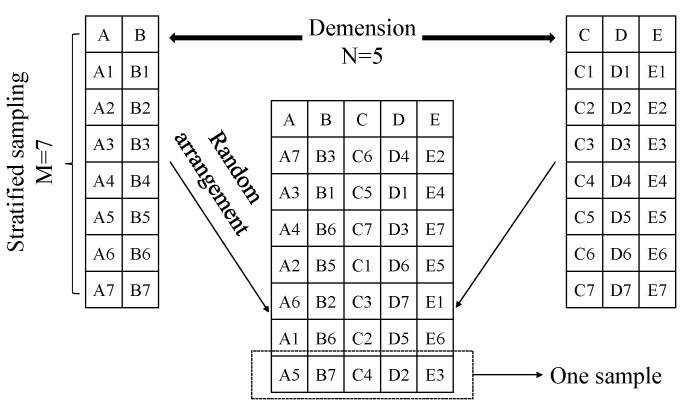
Latin hypercube sampling principle.

**Figure A3:**
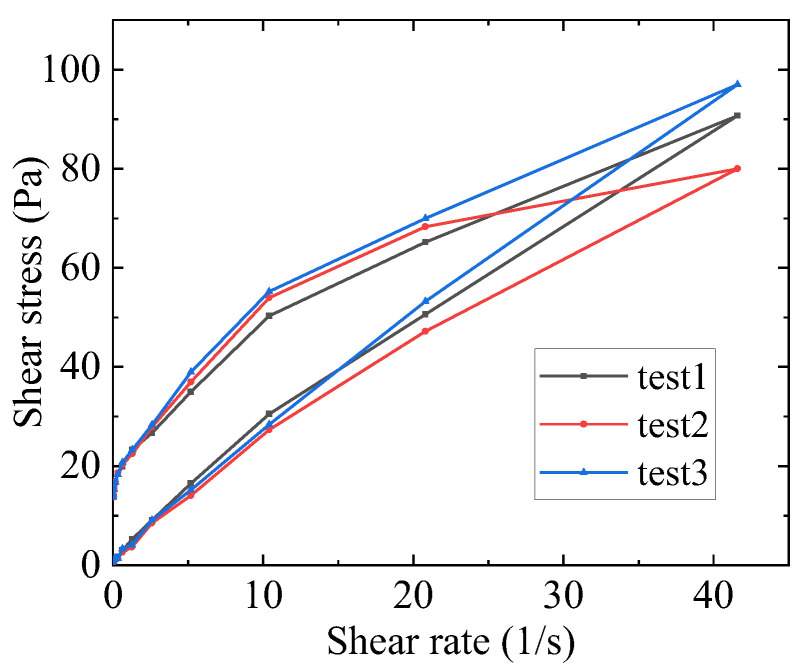
Hysteresis loop experiment results.

**Figure A4:**
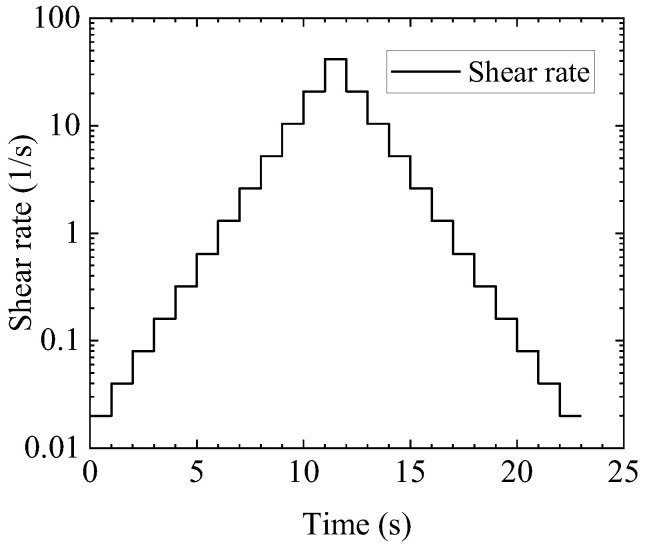
Shear rate and time relation in the hysteresis loop experiment.

**Figure A5:**
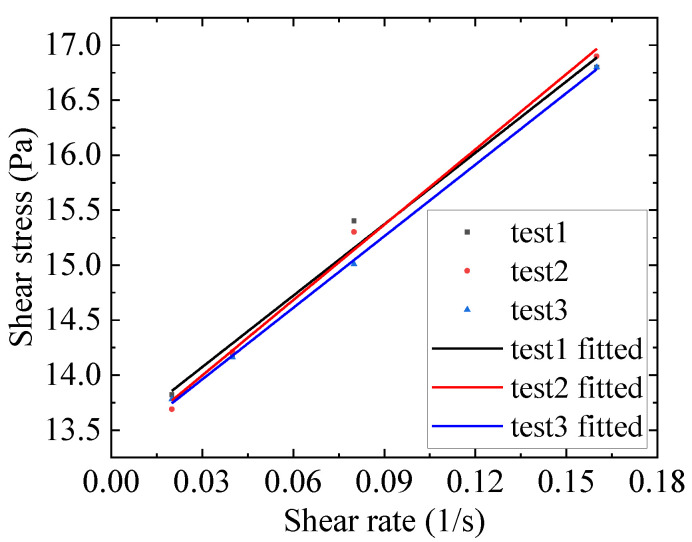
Initial linear stage fitting of the hysteresis loop experiment.

**Figure A6:**
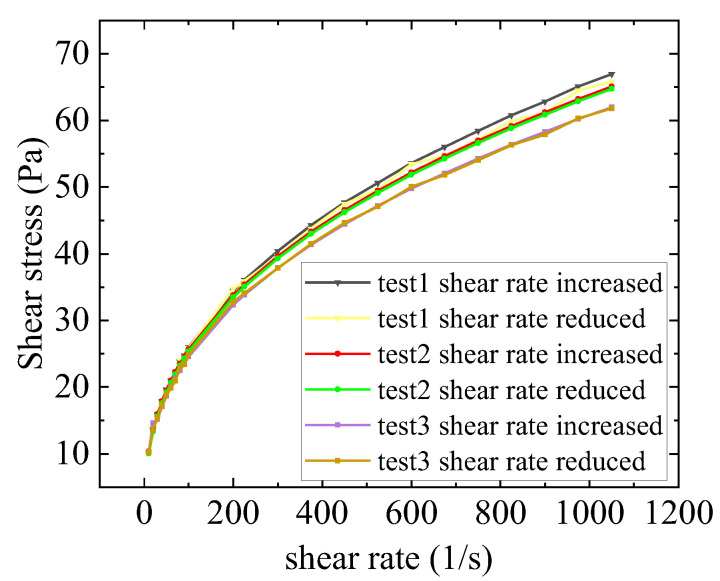
Hysteresis loop experiment after high-speed shearing.

**Figure A7:**
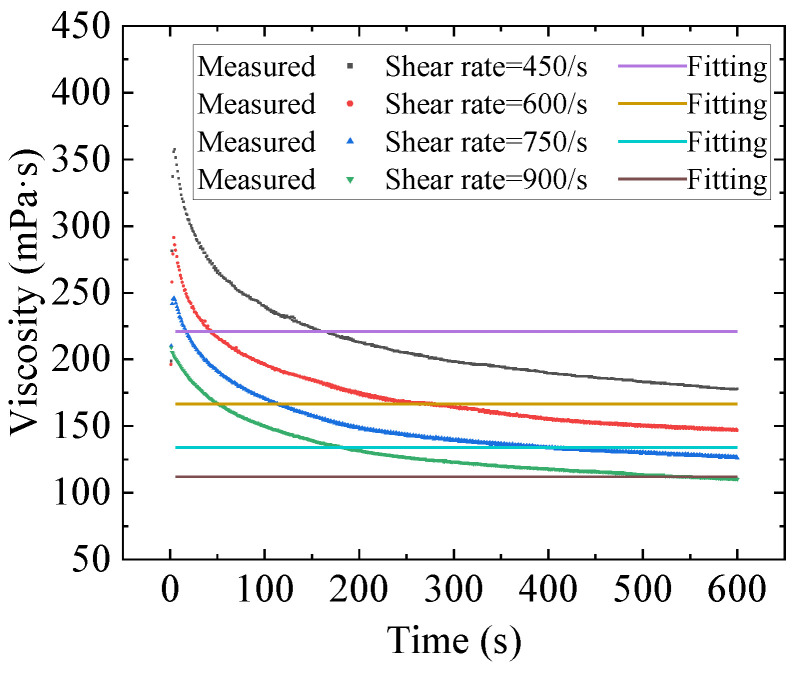
Toorman model transient viscosity fitting.

**Figure A8:**
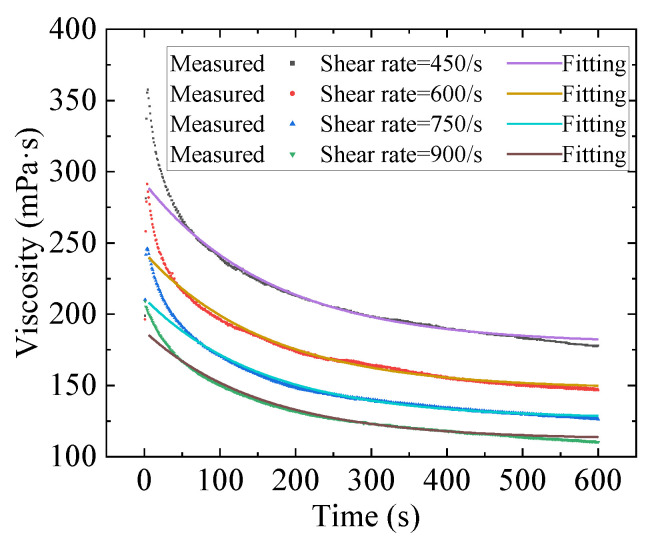
Houska model transient viscosity fitting.

**Figure A9:**
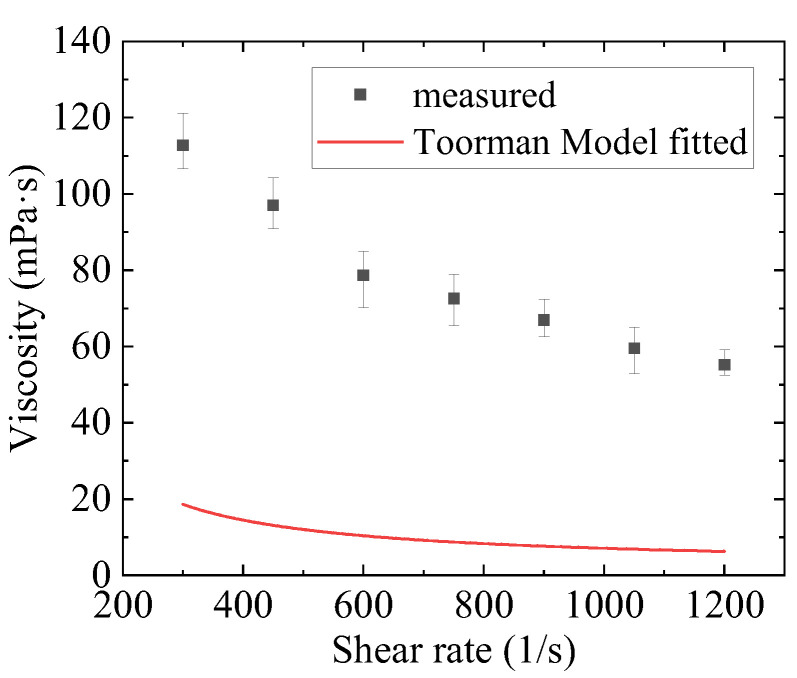
Toorman model steady-state fitting.

**Figure A10:**
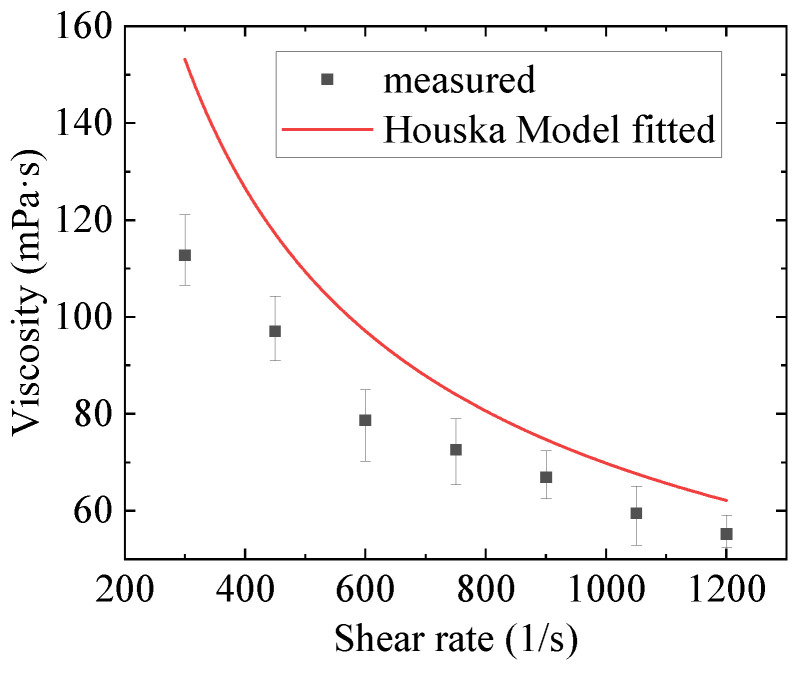
Houska model steady-state fitting.

**Figure A11:**
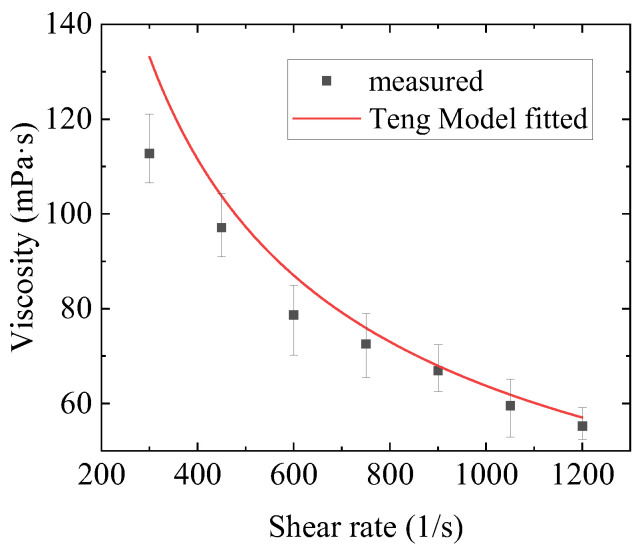
Teng model steady-state fitting.

**Figure A12:**
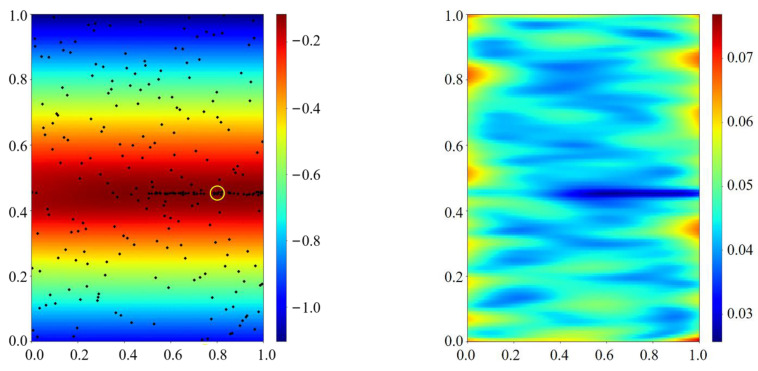
Two-parameter optimization process of a and b in the Toorman model.

**Figure A13:**
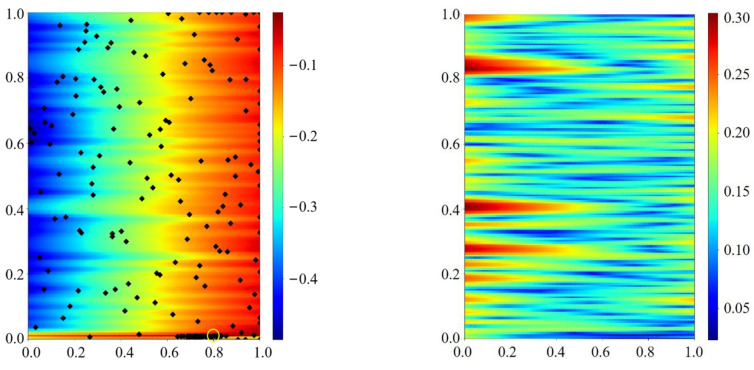
Two-parameter optimization process of a and b in the Houska model.

**Figure A14:**
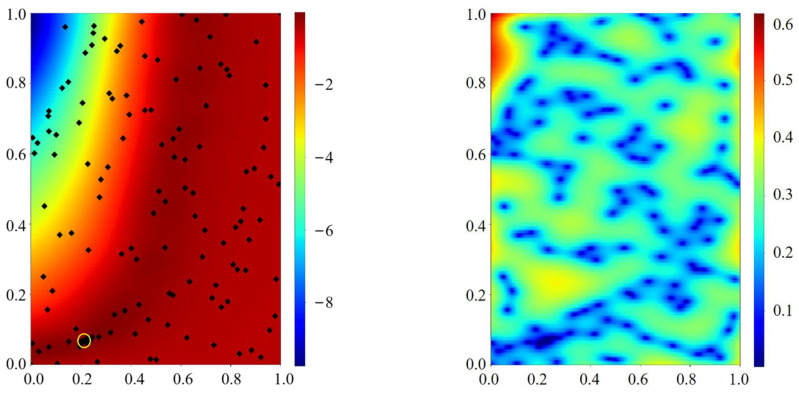
Two-parameter optimization process of Δk and m in the Houska model.

**Figure A15:**
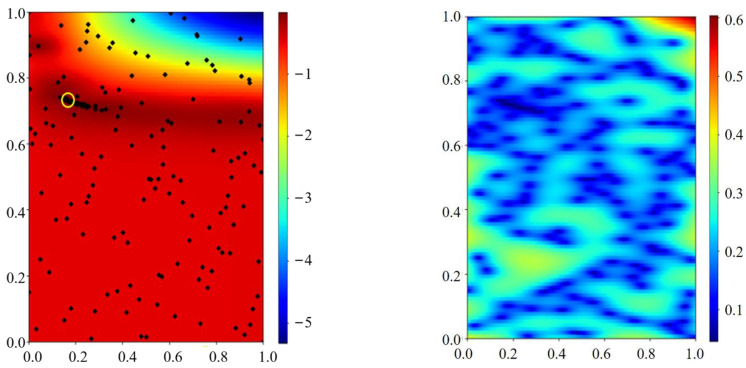
Two-parameter optimization process of Δk and $n_{2}$ in the Teng model.

## Data Availability

Not applicable.

## Appendix A

The composition of the gel is shown in Table A1.

**Table A1:** Composition of the prepared gel.

Composition	Mass Fraction
RP-3 kerosene	90%
Gelling agent (thixatrol ST)	5%
Additive (anhydrous ethanol)	5%

To prepare the kerosene gels, the kerosene was first heated to 45 °C in a water bath. By adding ethanol and Thixatrol ST to the kerosene, the kerosene gel formed immediately after dispersion with a high-speed disperser. To ensure uniform dispersion, the dispersion was continued at a constant temperature for 5 min to obtain a translucent milky white kerosene gel. Subsequently, the water bath was removed, and the gel was sealed and maintained at a constant temperature for 24 h before the subsequent rheological measurements were taken. The prepared gel is shown in Figure A1.

## Appendix B

All rheological measurements were performed using a Brookfield DV3THB rotational rheometer system, which was operated at a controlled rate, and the rotor models were CPE-40 and CPE-52. All experiments were carried out in a constant temperature water bath at 25 °C. The yield stress obtained through experimental measurements was related to the measurement method and measurement process [37]. Therefore, it is necessary to clarify the measurement method. In this paper, a direct measurement method was used to measure the yield stress, which measured the stress versus time directly under a low shear rate; then, the maximum stress was considered the yield stress [38,39,40,41]. To reduce the influence of the stress overshoot, a low-shear-rate rotor and low-speed conditions were selected for the yield stress test experiment; therefore, the CPE-52 rotor was selected with a rotation speed of 0.01 RPM and a shear rate of 0.02${\;s}^{-1}$. The curve of the stress versus time could be obtained through rheometer measurements, and then the yield stress value was determined. Regarding transient viscosity change measurements, the CPE-40 rotor was selected for the experiment on measuring the transient viscosity change. The transient process of the gel was measured by using shear rates of 900 $s^{-1}$, 750 $s^{-1}$, 600 $s^{-1}$ and 450 $s^{-1}$. The gel viscosity versus time was measured. To obtain the equilibrium viscosity value of the gel under different shear rates, the change in the viscosity with time was measured under a constant shear rate until the viscosity stabilized, and shear rates of 1200 $s^{-1}$, 1050 $s^{-1}$, 900 $s^{-1}$, 750 $s^{-1}$, 600 $s^{-1}$, 450 $s^{-1}$ and 300 $s^{-1}$ were selected. Finally, to obtain the rheological properties of the gel after high-speed shearing, the gel was pre-sheared at high speed in advance, and then its rheological properties were measured. In this paper, the shear stress–shear rate relationship measurement and hysteresis loop experiment were performed.

## Appendix C

### Appendix C.1. Pseudo-Code of the Bayesian Optimization

**Algorithm A1.** Bayesian optimization.
1: Initial sampling
2: Initial statistical model
3: For *n* = 1,2, do
4: Select new $X_{n+1}$ by optimizing acquisition function *f*
5: Query objective function to obtain $y_{n+1}$
6: Augment data $D_{n+1}=\left\{D_{n},\left(X_{n+1},y_{n+1}\right)\right\}$
7: Update statistical model
8: End for

### Appendix C.2. Bayesian Optimization Model

#### Appendix C.2.1. Gaussian Process Regression (GPR)

GPR is a machine learning method developed based on statistics and Bayesian methods. GPR does not need to explicitly specify the specific form of the function, and it is a nonparametric surrogate model. By assuming a priori that the objective function obeys a certain Gaussian process determined by the mean function and covariance function, the prior distribution of the function can be updated by using the posterior data, and the quadratic modelling model is finally obtained by continuously updating the posterior data.

A Gaussian process consists of a mean function and a covariance function and can be written in the following form:

(A1) $$f\left(x\right)\sim gp\left(m\left(x\right),k\left(x,x^{\prime }\right)\right)$$

where m(x) is the mean function of the function and $k\left(x,x^{\prime }\right)$ is the covariance function; that is, m(x)=E(f(x)), and $k\left(x,x^{\prime }\right)=E\left(\left(f\left(x\right)-m\left(x\right)\right)\left(f\left(x^{\prime }\right)-m\left(x^{\prime }\right)\right)\right)$. Generally, the covariance function is a positive definite matrix or a positive semidefinite matrix, which describes the correlation between the corresponding function values of different sample inputs, also known as the kernel function of the Gaussian process regression. It is difficult to choose a suitable prior function in the actual process. Therefore, the prior distribution of the mean function can be set to 0, and the posterior distribution of the mean function can be obtained through the modification of the sample data, which does not affect the accuracy of the posterior distribution. Since the Gaussian process can be regarded as a collection of random variables, any finite random variables satisfy the joint Gaussian distribution. Under the assumption of the zero mean, the prior distribution can be written as Equation (A2):

(A2) p(f|X,θ)=N(0,Σ)

where ***X*** is the sample set, ***f*** is the set of unknown functions *f*, Σ is the covariance matrix, $\Sigma_{i,j}=k\left(x_{i},x_{j}\right)$, and θ is the hyperparameter. The output value of the function corresponding to the sample points can be written in the form of a column vector: $y={\left[y_{1},y_{2}\ldots y_{n}\right]}^{T}$. The output and input can be written as a function: y=f(x)+ε, where ε is the noise value, assumed to exhibit a Gaussian distribution of the independent and identical distribution, that is, $\varepsilon \;\sim \;N\left(0,\sigma_{noise}^{2}\right)$. The prior distribution is shown in Equation (A3), and the likelihood distribution can be written as Equation (A4):

(A3) $$p\left(f|X,\theta \right)=N\left(0,\Sigma +\sigma_{noise}^{2}I\right)$$

(A4) $$p\left(y|f\right)=N\left(f,\sigma_{noise}^{2}I\right)$$

According to Equations (A3) and (A4), by using Bayes’ theorem, the marginal likelihood can be written as Equation (A5):

(A5) $$p\left(y|X,\theta \right)=\int^{\;}p\left(y|f\right)p\left(f|X,\theta \right)df=N\left(0,\Sigma +\sigma_{noise}^{2}I\right)$$

The hyperparameter θ is obtained by maximizing the marginal likelihood. First, the negative logarithm is taken to obtain the negative logarithmic marginal likelihood function shown in Equation (A6). Then, the partial derivative of L(θ) is calculated with respect to $\theta_{i}$. Finally, Newton’s method, gradient descent and other methods are used to iteratively update the result to obtain the hyperparameter value corresponding to L(θ):

(A6) $$L\left(\theta \right)=-\ln p\left(y|X,\theta \right)=\frac{1}{2}y^{T}C^{-1}y+\frac{1}{2}\text{ln}\left|C\right|+\frac{n}{2}\text{ln}2\text{π}$$

where $C=\Sigma +\sigma_{noise}^{2}I$, |C| is the determinant of C, and I is the identity matrix.

After the initial surrogate model is obtained from the initial sample points, it is necessary to predict the new sample point. For a given new sample $x_{new}$, the corresponding prediction value is $y_{predict}$, and the prior joint distribution formed by it and the original sample set is shown in Equation (A7):

(A7) $$\left[\begin{array}{c} y\\ y_{predict} \end{array}\right]\sim N\left(0,\left[\begin{array}{cc} K\left(X,X\right)+\sigma_{noise}^{2}I & K\left(X,x_{new}\right)\\ K\left(x_{new},X\right) & K\left(x_{new},x_{new}\right) \end{array}\right]\right)$$

where $K\left(x_{new},X\right)=K{\left(X,x_{new}\right)}^{T}$ is the 1 × *n* order covariance matrix between the test sample and the training set and $K\left(x_{new},X\right)=\left\{k\left(x_{1},x_{new}\right),k\left(x_{2},x_{new}\right)\ldots k\left(x_{n},x_{new}\right)\right\}$,$\;K\left(x_{new},x_{new}\right)$ is the variance of the test sample. According to Equation (A7), the conditional probability distribution, mean and variance of the predicted value can be obtained, as shown in Equations (A8)−(A10):

(A8) $$p\left(y_{predict}|X,y,x_{new}\right)=N\left({\bar{y}}_{predict},\mathrm{cov}\left(y_{predict}\right)\right)$$

(A9) $${\bar{y}}_{predict}=K\left(x_{new},X\right){\left[K\left(X,X\right)+\sigma_{noise}^{2}I\right]}^{-1}y$$

(A10) $$\mathrm{cov}\left(y_{predict}\right)=K\left(x_{new},x_{new}\right)+\sigma_{noise}^{2}I-K\left(x_{new},X\right){\left[K\left(X,X\right)+\sigma_{noise}^{2}I\right]}^{-1}K\left(x_{new},X\right)$$

where ${\bar{y}}_{predict}$ is the prediction mean and $\mathrm{cov}\left(y_{predict}\right)$ is the prediction covariance. In this way, the process of estimating the output distribution of the new point from the known sample point value is obtained.

When using Gaussian process regression, determining the kernel function is important. The kernel function used in this paper is formed by the combination of the Matern kernel and white noise kernel function. The expressions of a single kernel function and their combination form are shown in Equations (A11)–(A13):

(A11) $$k_{\mathrm{Matern}}\left(x_{i},x_{j}\right)=\frac{1}{\Gamma \left(v\right)2^{\nu -1}}{\left(\frac{\sqrt{2\nu }}{l}d\left(x_{i},x_{j}\right)\right)}^{\nu }K_{\nu }\left(\frac{\sqrt{2\nu }}{l}d\left(x_{i},x_{j}\right)\right)$$

(A12) $$k_{\mathrm{white}}\left(x_{i},x_{j}\right)=noise_{lever}\;\;\;\;\;if\;x_{i}=x_{j\;}\;\;\;\;\;else\;0$$

(A13) $$k\left(x_{i},x_{j}\right)=k_{\mathrm{Matern}}\left(x_{i},x_{j}\right)+k_{\mathrm{white}}\left(x_{i},x_{j}\right)$$

where ν is the smoothing parameter. Moreover, the larger the value of ν is, the smoother the process. Here, l is the scale parameter, $K_{\nu }$ is the modified Bessel function of the second kind, and $d\left(x_{i},x_{j}\right)$ is the Euclidean distance between two points. When ***x*** is a ***d***-dimensional vector, a scale parameter can be specified for each dimension. This approach also realizes automatic correlation determination for the kernel function. The larger the scale parameter is, the stronger the independence of the dimension. More detailed information concerning the Gaussian process regression is provided in Ref. [42].

#### Appendix C.2.2. Upper Confidence Bound Acquisition Functions

Cox et al. [43] introduced an algorithm called “Sequential Design for Optimization”, as shown in Equation (A14). They determined the upper bound of the confidence interval by adding the mean to the weighted variance:

(A14) UCB(x)=μ(x)+kσ(x)

where μ(x) and σ(x) are the mean and variance, respectively, and k is the variance weight. Generally, given an initial k value, the variance weight is gradually reduced in the optimization process, and the final acquisition function is completely determined by the mean value. During this process, the algorithm also gradually transitions from global exploration to local optimization.

### Appendix C.3. Sampling Theory

The samples in this paper are generated by using the Latin hypercube sampling method (LHS). This method was proposed by McKay et al. [44] and has strong space-filling ability and the ability to fit nonlinear responses. As shown in Figure A2, the LHS sampling method mainly consists of three steps—stratification, sampling, and out-of-order sampling. The basic principle is that if one needs to sample M sample points from the N-dimensional variable sample space, the sampling interval corresponding to each dimension in the N-dimensional variable is first divided into M nonoverlapping intervals. Moreover, a uniform distribution is generally considered, imparting each interval with the same probability. Then, a point is randomly selected from each interval of each dimension, and the order of the points is shuffled to form M vectors. The sample points drawn in this way are equally distributed over the entire random space. Compared with the Monte Carlo method, Latin hypercube sampling avoids the repetition and aggregation problems caused by direct random sampling. At the same time, it can ensure that the edge of the design space can be effectively sampled when there are few sample points.

## Appendix D

The hysteresis loop experimental curve of the gel is shown in Figure A3, and the variation in the shear rate is shown in Figure A4. Figure A3 reveals that the stress values of the gel under the same shear rate are not the same in the rising and falling stages of the shear rate, which is caused by the thixotropy of the gel. Indeed, the gel also shows shear-thinning properties. With an increasing shear rate, the slope of the stress–shear rate curve decreases gradually, which indicates a decrease in viscosity. Note also that when the shear rate is small, the stress measurement error is small. However, at a high shear rate, the stress has a large measurement error. This phenomenon is also due to the thixotropy of the gel. When the shear rate increases, the stress value changes dramatically during the sampling time.

The fitting results of the hysteresis loop experimental curve of the initial stage are shown in Table A2 and Figure A5, which can be used to obtain the value of C in the Toorman model.

**Table A2:** Linear fitting results of the cases.

Case	k	b	$R^{2}$
**#1**	21.65	13.42	0.985
**#2**	22.84	13.31	0.994
**#3**	21.70	13.31	0.999

## Appendix E

Upon high-speed shearing, the gel behaves as a power-law fluid, and the power-law model can be used to fit the stress–shear rate relationship. The power-law model is shown in Equation (A15), and the fitting parameters are shown in Table A3. The R-squared value reaches 0.997, which indicates high accuracy. According to the hysteresis loop experiment shown in Figure A6, the thixotropy of the gel almost disappears after high-speed shearing.

(A15) $$\tau =k{\dot{\gamma }}^{n}$$

**Table A3:** Power-law model parameters of the gels after high-speed shearing.

Fluid Processing	Fitted Power-Law Parameters		
	k	n	R-Squared
Kerosene gel processed by high-speed shear	4.14	0.395	0.997

## Appendix F

Figure A7 and Figure A8 show the fitting results in the transient process of the Toorman and Houska models.

Figure A9, Figure A10 and Figure A11 show the fitting results of the equilibrium viscosity of the Toorman, Houska and Teng models, respectively. Equations (A16)–(A18) are the corresponding equilibrium viscosity equations.

(A16)
$$\tau =\frac{\tau_{y}}{1+\frac{b}{a}\dot{\gamma }}+\left(\eta_{\infty }+\frac{C}{1+\frac{b}{a}\dot{\gamma }}\right)\dot{\gamma }$$

(A17)
$$\tau =\frac{a}{a+b{\dot{\gamma }}^{m}}\tau_{y}+\left(k+\Delta k\frac{a}{a+b{\dot{\gamma }}^{m}}\right){\dot{\gamma }}^{n_{1}}$$

(A18)
$$\tau =\frac{a}{a+b\phi^{m}}\tau_{y}+\left(k+\Delta k\frac{a}{a+b\phi^{m}}\right){\dot{\gamma }}^{n_{1}}$$

## Appendix G

For the Bayesian optimization process of the Toorman, Houska and Teng models, the following are the visualization fitting results of two parameters. In Figure A12, Figure A13, Figure A14 and Figure A15, the left panels show the optimized mean, and the right panels show the variance. The parameter range has been normalized. Figure A12 shows the two-dimensional visualization results of parameters a and b in the Toorman model. The parameter optimization ranges are as follows: b∈(0,0.2], a/b∈ [0,10]. The optimization process ultimately converges to b=0.162, a/b= 4.5. Figure A13 shows the two-dimensional visualization fitting results of parameters a and b in the Houska model. The parameter optimization ranges are as follows: b∈(0,0.1], a/b∈ [0,3]. The optimization process converges to b=0.0010, a/b= 2.4. Figure A14 shows the two-dimensional visualization fitting results of parameters Δk and m in the Houska model. The parameter optimization ranges are as follows: Δk∈ [0,100], m∈ [0,1]. The optimization process converges to Δk= 6.57, m= 0.20. Figure A15 shows the two-dimensional visualization fitting results of parameters Δk and $n_{2}$ in the Teng model. The parameter optimization ranges are as follows: Δk∈ [0,100], $n_{2}\in$ [0,1]. The optimization process converges to Δk= 17.54 and $n_{2}=$ 0.73. All convergence locations are indicated by the yellow circles in the figure.
